# Differential Attention Functioning in Pediatric Chronic Kidney Disease

**DOI:** 10.3389/fnhum.2022.897131

**Published:** 2022-06-24

**Authors:** Peter J. Duquette, Debbie S. Gipson, Stephen R. Hooper

**Affiliations:** ^1^Department of Physical Medicine and Rehabilitation, School of Medicine, University of North Carolina at Chapel Hill, Chapel Hill, NC, United States; ^2^Department of Pediatrics, Division of Nephrology, C.S. Mott Children’s Hospital, University of Michigan, Ann Arbor, MI, United States; ^3^Department of Health Sciences, School of Medicine, University of North Carolina at Chapel Hill, Chapel Hill, NC, United States

**Keywords:** attention dimensions, chronic kidney disease, end-stage kidney disease, attention, cognition

## Abstract

**Objective:**

To compare specific attention functions for school-age children with chronic kidney disease (CKD) to those of a typically developing control group.

**Methods:**

A cross-sectional study examined attention dimensions for children and adolescents with CKD (*n* = 30) in comparison to a typically developing control group (*n* = 41). The CKD group consisted of those receiving maintenance dialysis (*n* = 15) and those with mild/moderate CKD treated conservatively (*n* = 15). Measures aligning with Mirsky’s conceptual multidimensional model of attention were selected to compare groups across five dimensions of attention: Focus/Execute, Sustain, Stability, Shift, and Encode.

**Results:**

Significant group differences were revealed, with the CKD group performing worse than controls on the Focus/Execute, Sustain, and Encode dimensions. The CKD group also had a larger proportion of children with scores one standard deviation or more below the mean on the Shift and Encode domains, suggesting an at-risk level of functioning in these dimensions. Secondary analyses showed disease severity to be correlated with worse attention functions for children with CKD.

**Conclusion:**

Children with CKD may be vulnerable to subtle, specific deficits in numerous attention dimensions relative to their typically developing peers, particularly for those with more severe disease.

## Introduction

Chronic kidney disease (CKD) is a medical disorder that has been correlated with a number of neurodevelopmental concerns in childhood (Chen et al., 2018; Hooper et al., 2021; Johnson and Harshman, 2021), even in those with mild to moderate CKD (Hooper et al., 2011). One area of keen interest in pediatric CKD is attention functioning. While attention problems have been examined in the pediatric CKD literature, to date the findings have been mixed. Qvist et al. (2002) reported no group differences in attention abilities in their kidney transplant sample when compared with normative data, although a quarter of the sample continued to show attention deficits. In contrast, after adjusting for a variety of covariates, Mendley et al. (2015) found that 35% of their mildly impaired pediatric sample were at-risk for attention regulatory dysfunction, and that longer duration of CKD was significantly related to lower inhibitory control. Additionally, Mendley and Zelko (1999) noted improvements in attention and mental processing speed 1 year after transplant in their sample of nine children with CKD—which was consistent with the findings of Qvist et al. (2002). Most recently, functional neuroimaging studies have further unscored the potential neural mechanisms involved in attention-related difficulties (Harrell et al., 2021; Herrington et al., 2021) in pediatric CKD.

### Attention Multidimensionality

Most studies that have studied attention in pediatric CKD have examined it as a unidimensional construct, although several studies have shown children and adolescents with CKD to be at risk for different types of attention-regulatory abilities (e.g., selective attention; inhibitory control (Mendley et al., 2015; Yokoyama et al., 2020). Further, both animal and human research over the past two decades has increasingly suggested that attention is not a single entity but, rather, a multi-dimensional construct as illustrated by a number of different models proposing different attention functions (Posner and Petersen, 1990; Barkley, 1997; Mirsky et al., 1999; Park et al., 2009). Despite the various differences and nuances of each of these, most models of attention include some aspects of the following subcomponents: focused or selective attention, sustained attention, divided attention, and alternating or shifting attention.

One multidimensional model of attention that has a neurological and neuropsychological basis is the Mirsky Model (Mirsky et al., 1999). While the original model proposed by Mirsky and colleagues described several functions that were organized into a singular neurological system, a more recent examination of this model based on advancing neuroscience has identified multiple neurological systems underlying these attention components (Koziol et al., 2014); nonetheless, the various dimensions of attention remain viable neurocognitive constructs for examination. These dimensions correspond to the ability to attend selectively to the most relevant information (focus/execute), remain vigilant over an extended period of time (sustain), minimize fluctuations or variability in one’s focus (stabilize), process and output new information (encode), and change focus in a flexible or adaptive manner (shift). In the pediatric realm, this is important for understanding children with various kinds of attention problems (e.g., Attention-Deficit/Hyperactivity Disorder), including those with medical disorders that have been known to impact attention (e.g., spina bifida, pediatric cancer, epilepsy, chronic fatigue syndrome) (Dunn et al., 2003; Schatz et al., 2004; Burmeister et al., 2005; Haig-Ferguson et al., 2009). Examining different types of attention from a neuropsychological perspective also may provide translational indicators for a differential approach to intervention.

### The Current Study

Although attention has been identified as an area of concern in pediatric CKD, no previous studies have specifically examined attention in children with CKD using an empirically based, multi-dimensional model of attention. Using the neuropsychological model of attention of Mirsky et al. (1999), the current cross-sectional study compared children with CKD to a typically developing control group on specific attention dimensions. Based on previous literature, it was hypothesized that the CKD group would score lower than the control group across the specific attention dimensions, with a greater proportion showing attention dysfunction (defined as ≥1 standard deviation below the mean) across the five Mirsky dimensions. In secondary analyses, we anticipate that longer duration of CKD and more severe levels of CKD, as defined by GFR, also would be associated with lower scores on all attention dimensions.

## Materials and Methods

### Participants

Participants included children and adolescents with CKD (*n* = 30) and typically developing controls (*n* = 41) from a single medical center in the southeastern United States. Fifty percent of the CKD group consisted of individuals with End-Stage Kidney Disease (ESKD) receiving dialysis therapy (*n* = 15) and the other 50% included those with mild/moderate CKD managed with conservative therapies (*n* = 15). Among the CKD participants with ESKD, nearly all (*n* = 14) were receiving hemodialysis as opposed to peritoneal dialysis (*n* = 1). Inclusion criteria comprised those participants with CKD (defined as GFR ≤ 75 mL/min/1.73 m^2^), calculated by the Schwartz et al. (1976) formula, or dialysis-dependency for >3 months duration and chronological age between 6 and 18 years. The etiologies of CKD included obstructive uropathies/dysplasias (60%), glomerular disease (33%), and genetic disorders (7%). Approximately *40% of the CKD group had a history of grade retention, while 17% were receiving special education services.* Exclusion criterion included a history of prior kidney transplantation or the presence of a co-existing condition associated with central nervous system anomalies (e.g., closed-head injury, Down syndrome, Joubert syndrome). Control group participants (*n* = 41) were selected for participation if there was no prior history of chronic health conditions, head trauma, developmental disorder (e.g., intellectual disabilities, learning disabilities, Attention Deficit-Hyperactivity Disorder), neurological/psychiatric illness, or any current medication usage. All participants and their parent or legal guardian provided assent and consent prior to testing.

### Procedures

All participants were assessed as part of a larger pediatric nephrology study examining the neurocognitive effects of CKD and subsequent treatment modalities. The protocol and procedures were approved by the IRB. Standard procedures were in place so that participants in the CKD group underwent neuropsychological testing prior to any medical procedures (e.g., blood draws) to control for any negative reactions that could invalidate results. Similarly, testing did not coincide with dialysis procedures as that procedure occurred on an entirely separate day in an effort to avoid confounding of the pre- or post-effects of dialysis. Participants in the control group only underwent neuropsychological testing and were not subjected to any medical procedures.

Clinical variables included disease severity, as defined by GFR, duration of disease, age of onset, and co-morbid diagnoses (i.e., anemia, hypertension), and were obtained for participants in the CKD group. Anemia was defined as a hemoglobin level < 10.7 mg/dl, and hypertension was defined as measured blood pressure above the 95th percentile or the presence of antihypertensive therapy. Socioeconomic status was defined by maternal education which was nominally coded (1 = some high school, 2 = high school graduate or GED recipient, 3 = progress toward bachelor’s degree or completed associate’s degree, 4 = completed bachelor’s degree, 5 = completed graduate or professional degree).

### Measures

Measures were selected to align with the five-factor attention model posited by Mirsky et al. (1999): Focus/Execute—Gordon Diagnostic System (GDS) Mean Response Time (Gordon, 1986), Tower of London (TOL) Execution Time (Culbertson and Zillmer, 1998); Sustain—GDS Total Correct, Keith Auditory Continuous Performance Test (ACPT) Total Correct (Keith, 1994); Stability—GDS Correct Variability, ACPT Correct Variability; Shift—TOL Total Moves, Ruff Figural Fluency Test (RFFT) Perseveration (Ruff, 1996); and Encode—Wide Range Assessment of Memory and Learning (WRAML) Number/Letter and Finger Windows subtests (Adams and Sheslow, 1990). Means for each of the attention dimensions were calculated based on the age-based standard scores generated from each test variable. The Wechsler Abbreviated Scale of Intelligence also was used as an estimate of overall intellectual functioning (Wechsler, 1999).

### Data Analyses

Prior to conducting statistical analyses, scores for each of the dimensions of attention were calculated. Age-based standard scores (mean = 100, standard deviation = 15) were calculated from normative data for the following variables: TOL Total Moves, TOL Total Execution Time, GDS Total Correct, GDS Mean Response Time, GDS Correct Variability, ACPT Total Correct, WRAML Finger Windows, and WRAML Number/Letter. Means and standard deviations for the RFFT Perseverations and ACPT Correct Variability scores were generated from control group data for this age range so as to calculate standard scores for the entire sample. Scores were scaled such that higher standard scores indicated better performance for all variables. Once standard scores were generated for all variables, alpha coefficients were calculated as reliability indices to assess internal consistency within each attention dimension to determine if selected measures were at least moderately related to one another.

Preliminary analyses compared the CKD and control groups on the variables of age, sex, maternal education, and IQ to determine whether the groups differed systematically on any of these variables. *T*-tests were conducted to evaluate group differences between the CKD and control groups with respect to age and IQ. Pearson’s chi-square tests were run to compare groups on the variables of maternal education, and sex. If differences between the groups were evident, each variable was examined for its potential to confound results and whether to include it in subsequent multivariate analyses. Chronological age was chosen *a priori* as a covariate for all subsequent analyses given the wide age range in the entire sample and due to the conversion of RFFT and ACPT variables from raw scores to standard scores using all of the individuals in the control group.

For the primary analysis, scores on the five attention dimensions were compared between the CKD and control groups using MANCOVA and follow-up univariate procedures with a significant model. Estimates of effect sizes were calculated using partial eta squared (Cohen, 1988) and standard definitions for small (0.1–0.5), medium (0.6–0.13), and large (0.14 and above). The proportion of cases at-risk for attention dysfunction (i.e., one standard deviation or more below the normative mean) in the CKD and Control groups was compared for each of the five attention dimensions using chi-square.

Given the small sample size, a secondary exploratory univariate ANCOVA and corresponding chi-square tests for the at-risk status also were calculated to compare the attention dimensions across severity groups (i.e., control versus mild/moderate CKD versus ESKD). A Bonferroni correction was applied to adjust for the multiple comparisons. Additionally, correlations were obtained on CKD group data to examine the associations among the attention dimensions and selected disease-related variables including disease severity (i.e., GFR), age of CKD onset, and duration of CKD. Given the construction of several of the attention variables from sample-based data, chronological age was partialed out in the secondary correlation analysis.

## Results

### Preliminary Data Analyses

Internal reliability estimates using Cronbach’s alpha coefficient produced acceptable reliability for each of the five attention dimensions: Focus/Execute α = 0.56, Sustain α = 0.65, Stability α = 0.53, Shift α = 0.59, and Encode α = 0.67. Given this level of internal consistency, the attention dimensions were calculated as described above.

#### Demographic and Disease-Related Variables

As shown in Table 1, an independent samples *t*-test revealed no group differences on the mean age of participants, *t*(69) = 1.21, *p* = 0.23; nevertheless, as noted above, chronological age was used as a covariate in subsequent analyses given the wide age range of the sample and the use of the Control group to calculate standard scores for two attention variables. The CKD and Control groups differed significantly in their overall levels of intelligence based on the Full-Scale IQ from the WASI, *t*(69) = 6.99, *p* < 0.001, with the mean score for the CKD group falling at the lower end of the average range (Table 1); however, IQ was not co-varied in the analyses due to methodological arguments against its utility and the potential for overcorrection in the data (Dennis et al., 2009) and, in this instance, its likely association with our targeted attention outcomes. Chi-square tests revealed no differences in terms of sex distribution, χ^2^ = 0.53, *p* = 0.82, but significant group differences were evident for socioeconomic status as estimated by maternal education, χ^2^ = 20.06, *p* < 0.001. Thus, chronological age and SES were used as covariates in the MANCOVA.

**TABLE 1:** Characteristics for the CKD (*n* = 30) and Control (*n* = 41) groups.

Demographic variables	CKD group	Control group
Chronological age (years)	12.70 (3.32) (range: 6.45–18.84)	11.73 (3.36) (range: 6.11–18.94)
% Caucasian*	50.0	73.2
% Female	46.7	43.9
Maternal education***	2.93 (1.12)	4.15 (0.91)
WASI full scale IQ***	90.70 (15.59) (range: 64–127)	113.51 (11.94) (range: 72–138)
% Grade retention	40	0
% Special education	17	0
**CKD-related variables**		
CKD disease category	Mild/moderate = 15 (50%) ESKD = 15 (50%)	
Age of CKD onset (years)	5.11 (6.18) (range: birth–16)	–
Duration of CKD (years)	6.40 (4.73) (range: 0–17)	–
GFR	34.50 (27.37)	–
% Hypertensive	50.0	–
% Anemic	30.0	–

*p < 0.05; ***p < 0.001. Continuous variables presented as: mean (standard deviation); WASI IQ scores have a mean + 100 ± 15.

### Group Comparisons

Descriptive statistics for both groups are presented in Table 2. Results from the MANCOVA, controlling for age and maternal education, indicated significant differences between the CKD and control groups across attention dimensions as evidenced by a significant Wilks’ Lambda, *F*(5,63) = 2.48, *p* < 0.04. Follow-up univariate procedures indicated that the CKD group performed lower than the Control group on the dimensions of Focus/Execute, *F*(3,67) = 4.41, *p* < 0.007, Sustain, *F*(3,67) = 4.08, *p* < 0.01; and Encode, *F*(3,67) = 11.81, *p* < 0.001. Effects sizes for these group differences were large in magnitude. The CKD and Control groups did not differ significantly on the Stability (*p* < 0.08) and Shift (*p* < 0.18) dimensions, although medium effect sizes were present for each variable.

**TABLE 2:** Attention dimensions by group adjusted for chronological age and maternal education.

Attention dimension	CKD (*n* = 30)		Control (*n* = 41)			
	*M*	*SD*	*M*	*SD*	*F*-tests	Effect size
Focus/Execute	94.67	11.43	99.73	8.51	4.41**	0.17
Sustain	94.37	16.24	103.30	14.83	4.08**	0.15
Stability	87.13	18.35	96.88	16.26	2.38	0.10
Shift	91.50	11.02	95.52	11.88	1.67	0.07
Encode	88.92	9.35	99.33	10.09	11.81***	0.35

**p < 0.01; ***p < 0.001. Effect size presented as partial eta squared (0.01–0.05 = small; 0.06–0.13 = medium; 0.14+ = large). The attention dimensions have a mean + 100 ± 15, with higher scores reflecting more intact performance.

#### Severity Group Comparison

Secondary exploratory univariate analyses of variance (ANOVAs) were used to compare the control versus the mild/moderate CKD versus the ESKD groups on the attention dimensions (Table 3). Preliminary subgroup analysis did not reveal any differences between the two groups on chronological age, although this variable was covaried in these analyses given the wide age range. Additionally the three subgroups differed on maternal education (*p* < 0.004) and this variable was included as a covariate in the ANOVAs.

**TABLE 3:** Attention dimensions by severity subgroup adjusted for chronological age and maternal education.

Attention dimensions	Control (1) (*n* = 41)	Mild/moderate CKD (2) (*n* = 15)	ESKD (3) (*n* = 15)	*F*-tests df (4,66)	Pairwise comparisons
	***Mean (SD)***	***Mean (SD)***	***Mean (SD)***		
Focus/Execute	99.73 (8.51)	101.01 (12.43)	88.32 (12.33)	7.50***	3 < 1, 3 < 2
Sustain	103.30 (14.83)	101.54 (9.55)	87.19 (18.56)	5.04***	3 < 1, 3 < 2
Stability	96.88 (16.26)	95.41 (12.43)	78.84 (19.89)	3.57**	3 < 1, 3 < 2
Shift	95.52 (11.88)	92.78 (9.19)	90.22 (12.79)	1.26	
Encode	99.33 (10.09)	92.50 (6.48)	85.33 (10.56)	9.92***	3 < 1, 3 < 2

A modified Bonferroni correction was applied adjusting for the number of comparisons and degrees of freedom such that significance was set to **p < 0.01; ***p < 0.001. The attention dimensions have a mean + 100 + 15, with higher scores reflecting more intact performance.

Initial inspection of the mean scores across the five attention dimensions revealed the expected “stairstep” pattern (with the exception of the Mild/Moderate CKD Group being slightly higher than the Control Group on Focus/Execute), with the higher scores being demonstrated by the Control Group followed by the Mild/Moderate Group and then the ESKD Group. After adjusting for chronological age and maternal education, results of the univariate ANOVAs indicated that the groups differed significantly on four of the five attention dimensions: Focus/Execute (*p* < 0.001), Sustain (*p* < 0.001), Stability (*p* < 0.01), and Encode (*p* < 0.001). The groups did not differ significantly on the Shift attention dimension (*p* < 0.29). Follow-up pairwise comparisons for the significant overall findings revealed that the ESKD group performed significantly more poorly than the other two groups, with all scores falling within the bottom quartile. The Control group and Mild/Moderate CKD group performed similarly across the five attention dimensions ESKD, suggesting a possible dose effect related to severity.

### Proportion of Participants Performing > 1 Standard Deviation Below the Mean

Results from chi-square tests for the two group comparison (Table 4) with the Bonferroni correction (*p* < 0.04), indicated that the proportion of scores more than one standard deviation below the mean differed significantly between the CKD and Control groups on the attention dimensions of Shift, χ^2^(1) = 7.84, *p* < 0.005; and Encode, χ^2^(1) = 10.73, *p* < 0.001. The proportion of participants with attention dysfunction was similar between groups on the dimensions of Focus/Execute, χ^2^(1) = 1.52, *p* < 0.22; Sustain, χ^2^(1) = 2.46, *p* < 0.12; and Stability, χ^2^(1) = 1.75, *p* < 0.19.

**TABLE 4:** Proportion of participants by group deemed at-risk for attention impairment (i.e., scores ≥1 standard deviation below the mean).

Attention dimensions	CKD group (*n* = 30)	Control group (*n* = 41)	Pearson χ ^2^ (df = 1)
Focus/Execute	5/30 (17%)	3/41 (7%)	1.52
Sustain	9/30 (30%)	6/41 (15%)	2.46
Stability	10/30 (33%)	8/41 (20%)	1.75
Shift	10/30 (33%)	3/41 (7%)	7.84**
Encode	13/30 (43%)	4/41 (10%)	10.73**

A modified Bonferroni correction was applied adjusting for the number of comparisons and degrees of freedom such that significance was set to p < 0.03; **p < 0.01.

For the three-group comparison (Table 5) with the Bonferroni correction (*p* < 0.04) exploratory chi-square analyses revealed that the ESKD groups differed significantly in their proportions of cases deemed at-risk on all five dimensions, with the ESKD group having a significantly higher rate of individuals scoring in the at-risk range on each of these dimensions: Focus/Execute, χ^2^(2) = 9.85, *p* < 0.007; Sustain, χ^2^(2) = 12.23 *p* < 0.002; Stability, χ^2^(2) = 8.09, *p* < 0.02; Shift, χ^2^(2) = 7.84, *p* < 0.02; and Encode, χ^2^(2) = 15.30, *p* < 0.001. Consistent with the findings from the univariate ANOVAs, the ESKD group showed a significantly higher proportion of individuals falling in the at-risk range than either of the other two groups. The only attention dimension that deviated from that pattern somewhat was Shift, which showed similar rates of at-risk status for the CKD groups, and it was the only dimension where the Mild/Moderate group had a significantly higher proportion of individuals at-risk than the Control group.

**TABLE 5:** Proportion of participants by severity subgroup deemed at-risk for attention impairment (i.e., scores ≥1 standard deviation below the mean).

Attention dimension	Control group (1) (*n* = 41)	Mild/moderate CKD (2) (*n* = 15)	ESKD (3) (*n* = 15)	Pearson χ ^2^ (df = 2)	Subgroup comparisons
Focus/Execute	3/41 (7%)	0/15 (0%)	5/15 (33%)	9.85**	3 > 1, 3 > 2
Sustain	6/41 (15%)	1/15 (7%)	8/15 (53%)	12.23**	3 > 1, 3 > 2
Stability	8/41 (20%)	2/15 (13%)	8/15 (53%)	8.09*	3 > 1, 3 > 2
Shift	3/41 (7%)	5/15 (33%)	5/15 (33%)	7.84*	3 > 1, 2 > 1
Encode	4/41 (10%)	4/15 (27%)	9/15 (60%)	15.30***	3 > 1, 3 > 2

A modified Bonferroni correction was applied adjusting for the number of comparisons and degrees of freedom such that significance was set to *p < 0.04 for the Pearson χ^2^; **p < 0.01; ***p < 0.001. For the subgroup comparisons, significance was set to p < 0.05.

### The Relationship of Attention Dimensions to Selected Disease-Related Variables

The study was not powered to enter additional variables into the modeling, but it was important to determine the relationship of selected disease-related variables to the attention dimensions. After partialing out chronological age, correlations suggested significant associations between the Focus/Execute dimension and GFR. The magnitude of the relationship reached the moderate to strong range (*r* = 0.50). Similarly, the Sustain and Encode dimensions moderately and significantly correlated with GFR in the positive direction (*r* = 0.37 for both), indicating that better kidney functioning was associated with better attention in these three dimensions. Additionally, the Encode dimension moderately and significantly correlated with the duration of CKD, such that higher Encoding scores reflected a shorter disease duration (*r* = −0.35). The Focus-Execute Dimension did not significantly relate to age of onset or duration of CKD. The Stability and Shift dimensions of attention were not significantly related to any of the three disease-related variables. The co-morbidities of anemia and hypertension were not correlated with any of the attention dimensions, thus were not used in further analyses.

## Discussion

Although previous research has examined general cognition in pediatric CKD—including attention, less research has focused on the multidimensional components of specific neurocognitive functions. Furthermore, no previous studies have specifically looked at dimensions of attention in this population in comparison to one another. Attention is one of the most vital neurocognitive functions in that it factors into one’s ability to acquire, understand, retain, and regulate information in social and educational environments. In addition to learning and regulatory behaviors, it also has major implications among medical populations in terms of treatment compliance. The current study addressed this gap in the literature by comparing the performance of children with CKD to a typically developing comparison group using an empirically supported, multi-dimensional model of attention posited by Mirsky et al. (1999). Findings from this study provide one of the first glimpses at a possible attention profile in this population.

Findings suggested that specific aspects of attention may be vulnerable to the presence of CKD in childhood including the ability to concentrate attention resources (Focus/Execute), stay vigilant for an extended period of time (Sustain), and actively hold information in mind (Encode). The abilities to move from one salient aspect of a stimulus to another in a flexible, efficient manner (Shift) and to maintain consistency in response patterns over time (Stability) were not different between groups. The CKD and control groups also differed with respect to the proportion of participants showing at-risk status for attentional impairments (≥1 standard deviation below the mean) on the Shift and Encode attention dimensions, indicating that there is a significantly large number of children with CKD who will struggle with these types of attention. The proportion of participants in the CKD group who were at-risk for attention impairments also was similar in frequency to findings in at least one previous study (Qvist et al., 2002). Secondary data analyses across the attention dimensions showed the impact of disease severity, with the ESKD group performing more poorly and having a greater proportion of individuals at-risk for attention dysfunction across nearly all of the dimensions.

This finding implicating disease severity as impacting attention dimensions was further supported by the strong correlation between GFR and the attention dimensions of focus/execute, sustain, and encode. Interestingly, the stability and shift attention dimensions were not correlated with any of the CKD-related variables, perhaps secondary to the small sample sizes in the subgroups in this study and/or to some other unknown confounding factor. Other disease-related factors, such as duration of disease, may have small to moderate relationships with selected attention dimensions. While these associations warrant further investigation with a larger sample, the correlational findings were in the expected directions. For example, less disease severity related to better functioning in the Focus/Execute, Sustain, and Encode attention dimensions, while shorter disease duration related to higher scores on the Encode dimension. Identifying attention problems, in a more detailed fashion, may subsequently impact a child’s life course by guiding more specific types of educational and/or behavioral interventions (Groothoff, 2005), and future studies should examine whether this attention profile is similar to attention dimensions in other pediatric disorders (e.g., Neurofibromatosis Type I) (Prochnow et al., 2022).

The literature on attention in adults with CKD is broader, but has focused more on improvements in attention associated with the use of dialysis techniques or kidney transplant (e.g., Griva et al., 2004). Overall, the findings present a mixed picture in terms of the integrity of attention functions in this population. For example, Yount et al. (1998) utilized Mirsky’s model to examine data from 554 adults with ESKD. Similar to findings from our study, deficits in focused attention were found to be most strongly associated with CKD severity and lower education levels as well as older age. This pattern of findings also has been found for adult patients with ESKD who were awaiting a kidney transplant with dialysis-dependency (Lacerda et al., 2008), with the findings in the current study mirroring these findings as shown by our ESKD Group. Other adult studies on ESKD populations, however, have been inconclusive, with difficulties identified more frequently for processing speed and reaction time (Jassal et al., 2008) more so than for selective or sustained attention (Umans and Pliskin, 1998).

Several explanations exist for the group differences obtained in the current study. First, the effects of CKD on attention may be more prevalent in adolescence when academic demands in middle and high school require more sophisticated and intact attention. In this sense, task demands as a function of developmental stage may play a significant factor with encoding, focus/execute, and sustaining attention dimensions being most vulnerable early in the developmental process. A second explanation for the group differences relate to the effects of CKD severity. In this regard, children with the most severe forms of CKD who are more urgent candidates for a kidney transplant and receiving dialysis treatment may have even more significant attention difficulties, perhaps driven by both cognitive and emotional conditions. Although there were differences between the mild/moderate CKD and ESKD groups on selected attention dimensions, one might also suspect a higher potential for a more generalized impairment in cases of advanced kidney disease (i.e., increasing severity). Third, in all likelihood, there is a combination of developmental factors (e.g., changing task demands by age, treatment adherence, access to care and various social determinants of health), including psychosocial factors, that contribute to differential attention disruption. For example, as the various worries and concerns in both the child and caregiver regarding the child’s health increase with worsening kidney function, it is entirely possible that specific attention problems may be seen in the focus/execute and sustain functions. Further, there may be additional non-kidney related biological mechanisms in action, such as genetic predispositions (Verbitsky et al., 2017), that also could be interfering with the development of specific as well as general attention capabilities. Investigation of this complex of factors will require larger sample sizes and, perhaps, longitudinal designs.

Although this study examined a critically important avenue of assessment in pediatric CKD, the study did have a number of limitations that should be considered. First, the study included a small sample size, which precludes any broad generalization or interpretation of the results. Additionally, the small sample size reduced the power needed for inclusion of other covariates in the primary model, thus relegating key disease-related variables to secondary correlational analyses. Future research should endeavor to establish larger sample sizes through multisite collaboration to allow for inclusion of additional covariates (e.g., anemia, hypertension, seizures), thus resulting in more extensive analysis of potential predictors of attention function/dysfunction. This would assist in developing a predictive model of attention outcomes in CKD, giving pediatric nephrologists and other care providers of this population a valuable tool to guide collaborative, interdisciplinary care. Second, the study included a single control group. While this represented an appropriate initial comparison, it is not clear how this pattern of attentional dysfunction would compare or contrast to another pediatric chronic illness group, thus limiting the specificity of the findings. Indeed, this study also provided a signal regarding the importance of disease severity to attention function and a larger comparative group—even within the CKD spectrum (e.g., transplant versus dialysis versus mild/moderate CKD)—could be enlightening. Third, this study leaned on the Mirsky model of attention, but it is possible that another model of attention (e.g., Posner), or even a different operationalization of the Mirsky model using different neurocognitive measures, might lead to a different set of findings. Relatedly, while our internal reliability estimates of the attention dimensions were adequate for an initial study, the minimally satisfactory reliability of the dimensions warrants the need for improved measurement models with increased reliability (and thus validity) of specific attention functions. Finally, this study was decidedly cross-sectional in its design and, as such, could not address the critical issue of neurocognitive development within the context of a chronic illness and, in this instance, in the context of examining increasing disease severity in a continuous fashion. Future studies should endeavor to conduct longitudinal follow-up so as to provide better insight into the onset and development of specific and general attention impairments in CKD. This would allow for more targeted educational supports in terms of recognizing specific learning, behavioral, or emotional needs, as well as developing plans for extended absenteeism or attention-related fatigue related to CKD.

Although the sample size was small, results from the current study provide some of the most comprehensive findings to date on the differential attention functioning of children with CKD. Taken together, the results of this study suggest specific types of attention dysfunction, particularly in the focus/execute, sustain, and encoding, types of attention. This raises questions regarding the integrity of the underlying structure and function of frontal and prefrontal brain regions in the pediatric CKD population (Harrell et al., 2021; Herrington et al., 2021) in the development of these types of attention, with specific concerns related to the specific versus generalized impact of worsening disease severity on attention functions. These findings also raise questions regarding how and when neurodevelopmental trajectories of targeted brain regions may be affected by CKD (i.e., Are selected attention dimensions more vulnerable during their development with the onset and/or advancement of kidney disease?) and/or how other disease-related factors might impact these processes. Through long-term, systematic follow-up, the issue of differential attention impairments in CKD may be addressed in reference to the timeliness, type, and response to various interventions (e.g., behavioral, educational, pharmacological) for these children.

## Data Availability Statement

The raw data supporting the conclusions of this article will be made available by the authors, without undue reservation.

## Ethics Statement

The studies involving human participants were reviewed and approved by Institutional Review Board, University of North Carolina at Chapel Hill. Written informed consent to participate in this study was provided by the participants or their legal guardian/next of kin.

## Author Contributions

PD, DG, and SH contributed to the study design, collection of data, interpretation of the findings, manuscript preparation, and editing. PD and SH conducted the data analyses. All authors contributed to the article and approved the submitted version.

## Conflict of Interest

The authors declare that the research was conducted in the absence of any commercial or financial relationships that could be construed as a potential conflict of interest.

## Publisher’s Note

All claims expressed in this article are solely those of the authors and do not necessarily represent those of their affiliated organizations, or those of the publisher, the editors and the reviewers. Any product that may be evaluated in this article, or claim that may be made by its manufacturer, is not guaranteed or endorsed by the publisher.
